# Benefits of active life in student experiences during COVID-19 pandemic time

**DOI:** 10.3389/fpubh.2022.971268

**Published:** 2022-08-09

**Authors:** Saša Pišot, Ivana M. Milovanović, Darko Katović, Sunčica Bartoluci, Sandra S. Radenović

**Affiliations:** ^1^Institute for Kinesiology Research, Science and Research Centre Koper, Koper, Slovenia; ^2^Faculty of Sport and Physical Education, University of Novi Sad, Novi Sad, Serbia; ^3^Faculty of Kinesiology, University of Zagreb, Zagreb, Croatia; ^4^Faculty of Sport and Physical Education, University of Belgrade, Belgrade, Serbia

**Keywords:** epidemic emergency measures, the youth, eating habits, sleep habits, daily routine, studying

## Abstract

The first wave of the COVID-19 pandemic has led to extreme global consequences. In this paper, changes in the basic segments of students' everyday life and their subjective perception are investigated. The research was conducted in Croatia, Serbia and Slovenia. The application of the mixed method was performed to provide breadth and depth of understanding of students' responses to lifestyle changes. The research was conducted in two phases: a quantitative, using an online survey (*N* = 1,053), from April 15th to April 28th 2020, and a qualitative, using semi-structured interviews (*N* = 30), from June 10th to July 27th 2020. Students showed similar responses to the measures, but it turned out that the response in that population was different when their gender and study program were taken into account. The results suggest that students of study programs that are not “health-related” were more sensitive to change in habits than students of “health-related” study programs, but generally changes are visible in sleep patterns (going to bed late and waking up 60 to 80 min earlier). At the same time, the time spent in front of screens increased, from M = 4.49 (SD = 2.72) hours to M= 8.27 (SD = 3.44) hours during Covid-19, not only due to the transition to e-learning, but also due to a “stay at home” measure. Furthermore, students were less physically active, there was a decrease in exercise by 20 min (SD = 86.52) and a decrease in walking (M = 54 min, SD = 103.62) per day, and what is positive is that they were able to maintain the recommended amount of physical activity. The research contributes to the understanding of social consequences of extraordinary measures in students as young, healthy and highly educated social actors, as well as deeper insight into everyday strategies they undertake to counter or adapt to the new situation.

## Introduction

Coronavirus (COVID-19) causes severe acute respiratory syndrome coronavirus 2 (SARS-CoV-2) and was first reported in Wuhan, China, on December 31st 2019 (1–3). World Health Organization (WHO) declared the outbreak of the COVID-19 pandemic in March 2020. The pandemic caused a number of epidemiological, social, psychological, political and economic consequences for people around the world. Since the declaration of the pandemic on March 12th 2020 (3), until the end of this field research on July 27th 2020, 16.114.449 confirmed positive coronavirus cases have been recorded with 646.641 deaths reported by the WHO (4). As noted by Matthewman and Huppatz (5), the mortality rate from infections is largely correlated with age, and the negative physical effects of COVID-19 are most evident among the elderly population. When it comes to social impact, it is assumed that young people are most affected by “lockdowns” (5). Due to this, the focus of the research is the social impact of the COVID-19 pandemic on university students in Croatia, Slovenia and Serbia. The 2-month restrictive measures of the first pandemic wave probably had direct impact on the daily lives of most students and some adjustment was needed. In addition, a new life situation that a generation of students has never experienced before was in line with Zygmunt Bauman's “liquid life” theoretical framework, when the old balance between freedom and security was lost. The “new normal” turns life into a state of constant concern about the danger (natural and environmental disasters, random terrorist attacks, dangerous viruses that threaten people) that can happen unannounced at any time. Fear has become a name for insecurity because of the dangers that characterize our fluid modern age, with a lack of awareness of what poses a threat and our inability to determine what can and cannot be done against it (6). With fear of COVID-19, individuals are looking for some new goals to address in order to reduce excess insecurity, with personal security increasingly dominating daily plans. According to Bauman (7), the nature of *public* space and retreat into the *private* sphere could be seen, because concerned individuals are motivated by recommendations and instructions of government measures, barricade their homes and dream of the benefits of “fenced communities”. “Time of fear” also corresponds to the concept of “risk age” and/or “risk society”, as stated by Beck (8, 9). He mentions many aspects of the risk of global modern society, as well as the fact that new scientific discoveries and new technologies improve the quality of people's daily lives, but, on the other hand, produce and continue to create many uncertainties such as numerous health risks. These risks transcend national boundaries and do not differentiate between races, nations, social classes, social groups, gender, religion or religious affiliation (8, 9) because they become latent in the circumstances of the COVID-19 pandemic. The student generation (youth) in modern Western societies is vulnerable and enabled to reach individual maturity points fluidly and uncoordinated in time, to delay growing up and/or not be forced into certain adulthood patterns (10, 11) in postmodern society. Hence the new circumstances caused by the COVID-19 pandemic, the presence of “liquid life” in “liquid modernity” that affects rapid changes in habits and routines that have little chance of consolidating and settling and becoming a pattern, have fuelled uncertainty, insecurity and fear. This is also reflected in eating habits, sleeping habits, sports and recreational activities, which represent a significant part of the everyday life of young people (12). Individuals are the ones who have to find their own ways to deal with the anxiety and fear that is endemic to “liquid life”.

Relying on the referential theoretical framework (7–9), as well as previously published papers on compatible topics (12, 13), this paper is based on the results of field research of everyday student practices in three European countries: Croatia, Serbia and Slovenia. Namely, this is part of a larger study we conducted in the first wave of the pandemic from 15th to 28th April 2020, on the population of citizens aged 18 to 82 (*N* = 4,018) in nine European countries (12). When processing the data, we noticed greater similarities (see Table 1) of epidemiological measures in Croatia, Slovenia and Serbia and the limitation in the implementation of study programs in isolation as well as the similarity of the epidemiological situation (number of infected/100,000 inhabitants, number of deaths/100,000 inhabitants) in comparison with the epidemiological situation and emergency measures in other European countries covered by the study. Consequently, these three societies represent the spatial framework of this research. Furthermore, although social grouping can be based on different bases (such as gender, age, region of residence, etc.), for the purposes of this research we have chosen active university education as a key feature of grouping. Hence, the demographic feature – generational affiliation – in conjunction with the participation in a certain educational sphere, prompted us to single out students for research purposes as a relatively autonomous social group. We also assumed that the mentioned generational and educational affiliation conditions similarities in the domain of lifestyles, economic dependence or independence and other features of the “transition to adulthood”. In the current public health crisis, this can lead to an unequal social position of the student population, which is present in both private and public spheres of their social life, which is why we assume that the student population is socially highly affected by government measures and “lockdowns” and reduced freedom of movement. By analyzing the restrictive measures in these societies, it is obvious that the threat of pandemic spread was taken seriously in all three countries and their governments reacted quickly by introducing these measures, between 16th and 20th of March 2020. These measures included the closure of educational institutions (schools, colleges, dormitories). A new set of measures followed to ensure physical distance (2 meters indoors, 1 meter outdoors). The mentioned measures prohibited all activities involving a large number of people in close contact and gatherings of more than 5 people. All activities that were not necessary were canceled, but many facilities and shops remained open to ensure the usual delivery of food and goods. Studying and communicating with the outside world have been moved to online platforms i.e., online teaching and social networks, while restricting movement has restricted individual freedom, that lasted until the 1st week of May 2020.

**Table 1:** Emergency measures in Croatia, Slovenia and Serbia in the first wave of COVID-19 (review of everyday life and university education).

**Country**	**The first declared case of COVID-19**	**When did the competent authorities in your country start to apply the measures?**	**What are these measures in particular society?**	**What are the measures in the field of higher education?**	**When did the “easing of measures” begin?**
Croatia^*^	25. of February 2020	19. of March 2020	Closing public life, distance limitation, ensured normal supply of food and goods;	Switching to online teaching, Eviction of students from dormitories	3. of May 2020
Slovenia^*^	04. of March 2020	12. of March 2020	State of emergency, locking of kindergartens, schools, colleges, playgrounds, (with the exception of) sports gyms	Switching to online teaching, Eviction of students from dormitories	6. of April 2020
Serbia^*^	6. of March 2020	16. of March 2020.	State of emergency; curfew 18:00-5:00 20. of March 2020 15:00-5:00	Switching to online teaching, Eviction of students from dormitories	21. of April 2020

* NIJZ -National institute of public health Slovenia; *Data of Croatia on official web site: https://www.koronavirus.hr/; *Data of Slovenia available on official Slovenian government web site: https://www.gov.si/en/topics/coronavirus-disease-covid-19/; *Data of Serbia available on official web site: https://covid19.rs/.

Assuming that the proclamation of the COVID-19 pandemic and the introduction of extraordinary epidemiological measures directly affected students' daily lives, the main goal was to investigate changes in daily practices, habits and routines of students in different study programs and the ways in which they responded, reacted and adapted to new circumstances.

In addition, we assumed that possible changes in differences arose as a result of the study or the so-called “high awareness of healthy lifestyle practice”. As we have witnessed an acute decline in physical activity (12, 14, 15) caused by movement restrictions in the first wave of the pandemic, this could be especially true for those students who lost access to campus recreational facilities and social support for physical activity after university closures nationwide. Based on evidence of positive effects of physical activity on stressful life events (16), we assume that students of health majors (study programs) will respond more positively to sudden changes in daily practice, habits and routines than others.

In this regard, it was necessary to determine the extent to which students of study programs, which we divided into health-related study program group, SPG1 (study programs of sports and physical education, kinesiology, medicine, health) and those from other study programs – study program group, SPG2, managed to maintain their daily life habits and routines, in extraordinary social circumstances. Specific objectives included research into (possible) changes in the domain of:

- sleeping habits;
- eating habits (frequency and size of meals, consumption of fast, unhealthy or high-calorie foods; consumption of alcohol and smoking);
- awake time related to physical activity and inactivity, time spent in front of the screen as the dominant activity in a sitting position;
- new routines or routines that occurred when others were discontinued.

As habits repeat relatively automatically, with little variation, but at the same time, as habits interact with time, other people and context, and therefore are not performed in the exact same way over and over again (17), we also focused on routines as regular, less or more immutable procedures, common, prescribed, or daily, both business and personal (18). The study of daily routines as relatively fixed time patterns of consecutive activities in which one participates during a typical day (19, 20) was divided into habits (sleeping and eating), and physical activity and other routines, based on the attitude that people differ in routines, which are defined as the degree to which people are “motivated to keep everyday events in their lives in relatively unchanged and orderly patterns” (21). As we stated, for the purposes of analysis students were divided into two different study program groups: SPG1 - sports or applied kinesiology study programs, medicine; and study program group, SPG2 - other study programs such as: natural and technical sciences; humanities and social sciences; and the rest, in order to establish various everyday life circumstances, based on a particular study program. We assumed that there is a difference in the domain of life habits and lifestyles of students/members of two different groups of study programs.

## Methods

Mixed (quantitative and qualitative) field research was conducted in three European countries (Croatia, Slovenia, Serbia), with the aim of observing and analyzing changes in daily life habits and routines of university students (graduate and postgraduate programs), during the first wave of emergency epidemiological measure due to COVID-19. The use of the mixed method expanded the scope of the research by using research components of the questionnaire and semi-structured interview type, and additionally provided an explanation of the results of the quantitative method with the results of the qualitative method.

### Sample

The sample consists of students from the University of Zagreb, Split, Rijeka and Osijek in Croatia, the University of Novi Sad and Belgrade in Serbia, and the Universities of Ljubljana, Maribor, Primorska, Alma Mater Europaea and the Faculty of Physiotherapy in Slovenia, who filled in an online questionnaire (12) (*N* = 1053, n _(Male)_ = 387, n _(Female)_ = 666; M _age(Male)_ = 22.04, SD _age(Male)_= 2.74, Min _age(Male)_ =18, Max _age(Male)_ = 34; M _age(Female)_ = 21.80, SD _age(Female)_ = 2.39, Min _age(Female)_ =18, Max _age(Female)_ =34).

We assumed that there are possible differences in lifestyle changes and coping strategies between students of different study programs. That is the reason why the sample was divided into two different study program groups and two gender samples (see Table 2).

**Table 2:** Sample structure of respondents – survey.

**Gender**	**Study program**	***n***	**%**
F	SP1	182	27.3
	SP2	484	72.7
	Total	666	63.3
M	SP1	167	43.1
	SP2	220	56.7
	Total	387	36.7

*n, the number of entities in the subsample; %, relative frequency*.

The sub-sample of entities used in the qualitative research consists of students from these universities who participated in online questionnaires and who were willing to participate in semi-structured interviews (Table 3). The interview was conducted by three sociologists, who are also members of the consortium “Everyday life practices-ELP COVID-19” (12).

**Table 3:** Sample structure of respondents – interview.

**Gender**	**Study program**	***n***	**%**
F	SP1	7	46.7
	SP2	8	53.3
	Total	15	
M	SP1	7	46.7
	SP2	8	53.3
	Total	15	

*n, the number of entities in the subsample; %, relative frequency*.

### The sample of variables

The sample of variables of this study consists of a subset of 14 questions taken from the study (12) presented as the resulting difference (delta) between the results of variables recorded at the time of emergency epidemiological measures compared to the results of variables that subjects had before the introduction of emergency epidemiological measures and 2 category variables: Study program group, (SPG1, SPG2) and gender (Female, Male).

Variables can be grouped according to the types of changes according to:

- Changes in sleeping habits: deltaQ2a (going to bed), deltaQ2b (waking up), deltaQ3 (sleeping time).
- Changes in physical activity and inactivity: deltaQ4 (inactivity time), deltaQ10 (walking for transport) and deltaQ11 (exercise/recreation), deltaQ13 (moderate or high physical activity).
- Changes in time spent in front of the screen: deltaQ6 (time in front of the screen), deltaQ7c (time on the computer), deltaQ8a (time in front of the screen for learning).
- Variables of regular meals (Q17a), consumption of unhealthy foods (Q17c), alcohol (Q17d) and smoking (Q17e), which belong to the group of variables for the analysis of eating habits (current eating habits compared to the previous usual period) are shown on Likert scales (1–5, much less, less, same, a little more, a lot more).

### Questionnaire

The questionnaire was distributed in the form of an online survey in Croatian, Serbian and Slovenian in the period from 15^th^ to 28^th^ of April 2020. It contains a total of 24 questions with customized parts of the questionnaire (SIMPAQ – Simple Physical Activity Questionnaire) (22), which indicate data on sleep time, physical activity time (TA) and inactivity time before and during emergency epidemiological measures related to COVID-19 pandemic.

Questions and scales for assessing eating habits and together with the adapted scale for assessing changes in eating and other health habits (alcohol and smoking) were taken from EHIS (European Health Interview Survey, National Institute of Public Health in Slovenia [NIJZ], 2007). Survey data were collected and analyzed in accordance with the General Data Protection Regulation (GDPR).

### Semi-structured interviews

Qualitative data were collected through thirty semi-structured interviews with university students from Croatia, Serbia and Slovenia (15 male and 15 female students), from June 10^th^ to July 27^th^ 2020. The interviews were structured around two main topics:

- changes in daily life routines and habits during anti-COVID-19 measures regarding sleep habits, eating habits, waking time related to physical activity, sedentary time and time spent in front of a screen;
- psycho-social changes due to the impact of restrictive measures against COVID-19 (personal relationships, feelings, coping with new learning regimes and perceptions of the future) that were not the subject of analysis in this paper.

The questions asked to the interviewees within the mentioned topics were aimed to indicate more deeply and specifically whether there have been any changes at all in the domains of sleep, nutrition, physical activity and time spent in front of screens. Detailed descriptions of changes in daily routines, conditioned by the establishment of isolation, and then the creation of “new routines” as the isolation lasted longer and longer created the basis for questions about the interviewees' perception of what psycho-social consequences these changes caused in their lives. Researchers did not encounter ethically questionable situations during the research. Anonymity is guaranteed to all participants, and each interviewees was given a number and was categorized according to the type of the study programme. All interviewees agreed to participate in the research (each of them signed an agreement to participate) and were informed about all the conditions under which the data obtained during the research will be used. Interviews were recorded using a dictaphone or a smartphone app. All recordings are stored in private researchers' databases.

The average age of the interviewees was 21.86 years (SD = 2.06). The average duration of the interview was 23:37 min. The shortest interview was 8:38 and the longest was 50:53. The duration of the interview was related to the type of interviewees (dominated by moderately honest and completely honest type of interviewee), age of the interviewee, year of study, study program, but also the need and willingness of the interviewee to speak more or less openly about their personal experience of the emergency measures during the COVID-19 pandemic. Most of the interviews were conducted live (in offices or other places), and seven (6) of them were conducted by video call (MS Teams, Zoom), because the current circumstances of the daily life of the interviewees required such interviews.

## Results

Descriptive parameters (Mean, Median, Mode, Standard deviation, Minimum, Maximum, Range, Skewness, Kurtosis) were calculated for all quantitative variables. The normality of the distribution was tested by the Shapiro-Wilk test and the Q-Q plot. Descriptive parameters were calculated separately for the male subsample of students and female subsample of students of both study program groups (SPG1, SPG2) (see Tables 4, 5).

**Table 4:** Descriptive statistic for study program groups (SPG1 and SPG2) – male students.

**Var**.		**Valid (*n*)**	**Median**	**Mean**	**Std**.	**Skew**.	**Kurt**.	**Shapiro–Wilk (*p*)**	**Range**	**Min**.	**Max**.
deltaQ2a	SPG1	167	1.000	1.079	1.555	−0.218	−0.131	0.007	8.000	−3.000	5.000
deltaQ2a	SPG2	220	1.000	1.068	1.736	−0.607	1.989	<0.001	12.500	−6.500	6.000
deltaQ2b	SPG1	167	2.000	1.715	1.682	−0.252	1.949	<0.001	12.000	−4.000	8.000
deltaQ2b	SPG2	220	1.500	1.360	1.951	−0.767	3.854	<0.001	15.000	−8.000	7.000
deltaQ3	SPG1	167	−0.500	−0.687	1.488	−0.985	2.120	<0.001	9.000	−7.000	2.000
deltaQ3	SPG2	220	0.000	−0.443	1.426	−0.180	0.979	<0.001	9.000	−5.000	4.000
deltaQ4	SPG1	167	3.000	3.428	2.596	0.096	0.653	<0.001	15.000	−5.000	10.000
deltaQ4	SPG2	220	2.000	2.784	3.974	0.571	0.941	<0.001	22.000	−7.000	15.000
deltaQ10	SPG1	167	−40.000	−50.060	97.194	0.547	3.227	<0.001	680.000	−300.000	380.000
deltaQ10	SPG2	220	−40.000	−49.445	106.260	−0.707	3.653	<0.001	840.000	−600.000	240.000
deltaQ11	SPG1	167	−30.000	−39.407	96.458	−0.355	0.845	0.033	600.000	−380.000	220.000
deltaQ11	SPG2	220	0.000	−13.050	77.113	−0.585	6.207	<0.001	670.000	−370.000	300.000
deltaQ13	SPG1	167	0.000	20.868	74.278	0.974	16.214	<0.001	890.000	−420.000	470.000
deltaQ13	SPG2	220	0.000	17.373	113.356	3.951	46.686	<0.001	1,618.000	−478.000	1,140.000
deltaQ6	SPG1	167	3.000	3.428	2.406	0.078	1.752	<0.001	17.000	−6.000	11.000
deltaQ6	SPG2	219	3.000	3.486	3.177	0.332	0.837	0.003	21.000	−6.000	15.000
deltaQ7c	SPG1	167	1.000	1.743	2.098	1.095	1.869	<0.001	12.000	−3.000	9.000
deltaQ7c	SPG2	220	2.000	2.466	2.765	0.785	1.766	<0.001	19.000	−7.000	12.000
deltaQ8a	SPG1	167	0.500	0.641	1.769	−0.230	2.063	<0.001	13.000	−7.000	6.000
deltaQ8a	SPG2	220	1.000	1.175	2.795	0.631	4.478	<0.001	23.000	−9.000	14.000
Q17a	SPG1	166	3.000	3.277	1.019	−0.057	−0.213	<0.001	4.000	1.000	5.000
Q17a	SPG2	215	3.000	3.344	0.929	0.216	−0.475	<0.001	4.000	1.000	5.000
Q17c	SPG1	164	3.000	2.817	1.147	0.044	−0.834	<0.001	4.000	1.000	5.000
Q17c	SPG2	215	3.000	2.781	1.141	0.057	−0.707	<0.001	4.000	1.000	5.000
Q17d	SPG1	98	1.000	1.714	0.931	1.231	0.970	<0.001	4.000	1.000	5.000
Q17d	SPG2	160	2.000	2.025	1.154	0.795	−0.498	<0.001	4.000	1.000	5.000
Q17e	SPG1	37	3.000	2.676	1.156	0.001	−0.670	0.002	4.000	1.000	5.000
Q17e	SPG2	76	3.000	2.526	1.227	0.182	−0.852	<0.001	4.000	1.000	5.000

**Table 5:** Descriptive statistic for study program groups (SPG1 and SPG2) – Female students.

**Var**.		**Valid (*n*)**	**Median**	**Mean**	**Std**.	**Skew**.	**Kurt**.	**Shapiro–Wilk (*p*)**	**Range**	**Min**.	**Max**.
deltaQ2a	SPG1	182	1.250	1.330	1.643	−0.212	0.764	0.003	10.750	−4.000	6.750
deltaQ2a	SPG2	484	1.000	1.179	1.727	−0.625	2.757	<0.001	15.500	−9.500	6.000
deltaQ2b	SPG1	182	2.000	2.059	1.883	0.093	0.734	0.034	11.500	−3.000	8.500
deltaQ2b	SPG2	484	2.000	1.774	1.708	−0.129	1.306	<0.001	12.500	−4.500	8.000
deltaQ3	SPG1	182	−1.000	−0.826	1.499	−0.332	0.426	0.001	8.500	−5.500	3.000
deltaQ3	SPG2	484	−1.000	−0.854	1.741	−0.112	1.345	<0.001	12.000	−7.000	5.000
deltaQ4	SPG1	182	3.000	3.805	3.398	0.305	0.486	<0.001	19.500	−6.000	13.500
deltaQ4	SPG2	484	3.000	2.843	3.350	−0.145	0.905	<0.001	25.000	−12.000	13.000
deltaQ10	SPG1	182	−60.000	−74.363	107.216	−0.361	0.959	<0.001	690.000	−390.000	300.000
deltaQ10	SPG2	484	−40.000	−51.217	102.987	−0.300	2.653	<0.001	840.000	−420.000	420.000
deltaQ11	SPG1	182	−30.000	−49.150	108.031	−0.192	2.482	<0.001	810.000	−360.000	450.000
deltaQ11	SPG2	484	0.000	−7.378	74.320	−0.647	4.245	<0.001	700.000	−400.000	300.000
deltaQ13	SPG1	182	0.000	26.132	77.829	1.478	8.434	<0.001	740.000	−320.000	420.000
deltaQ13	SPG2	484	0.000	16.665	65.800	−0.253	11.740	<0.001	735.000	−375.000	360.000
deltaQ6	SPG1	182	4.000	3.880	2.828	0.623	1.588	<0.001	18.000	−4.000	14.000
deltaQ6	SPG2	484	4.000	4.001	3.207	−0.058	1.704	<0.001	25.000	−11.000	14.000
deltaQ7c	SPG1	182	1.500	1.808	2.214	1.211	3.682	<0.001	17.000	−5.000	12.000
deltaQ7c	SPG2	483	2.000	2.513	3.131	0.374	2.031	<0.001	26.000	−9.000	17.000
deltaQ8a	SPG1	182	1.000	0.997	2.165	0.365	2.490	<0.001	16.000	−6.000	10.000
deltaQ8a	SPG2	483	2.000	1.848	3.204	−0.466	1.703	<0.001	25.500	−12.000	13.500
Q17a	SPG1	179	3.000	3.291	1.003	−0.272	−0.346	<0.001	4.000	1.000	5.000
Q17a	SPG2	475	3.000	3.475	1.033	−0.311	−0.306	<0.001	4.000	1.000	5.000
Q17c	SPG1	179	3.000	2.939	1.142	−0.084	−0.697	<0.001	4.000	1.000	5.000
Q17c	SPG2	476	3.000	2.981	1.228	−0.108	−0.997	<0.001	4.000	1.000	5.000
Q17d	SPG1	102	1.000	1.833	1.025	1.017	0.290	<0.001	4.000	1.000	5.000
Q17d	SPG2	342	1.000	1.874	1.096	1.031	0.067	<0.001	4.000	1.000	5.000
Q17e	SPG1	50	3.000	2.360	1.321321	0.510	−0.701	<0.001	4.000	1.000	5.000
Q17e	SPG2	188	2.000	2.436	1.333	0.447	−0.948	<0.001	4.000	1.000	5.000

Analyzing the parameters of the form of distribution, homogeneity of variance, normality of distribution of residual values, and balance of subsamples, it was found that the necessary prerequisites for using the parametric method of differences were not met so Mann Whitney *U* test was used to determine differences between two samples. The analysis was performed using the R (23) software environment for statistical computing and graphics, using the “psych” (24) and “dplyr” (25) packages.

For the empirical material collected in the interviews, qualitative analysis software was used (26) NVivo 12 for data storage, transcription for recording connections, notes and arrangements of codes and nodes. In addition, the researchers performed data analysis, imaginative research and thinking. Interviews were conducted according to an agreed protocol. The initial code tree also tracked blocks of protocol questions, as well as additions agreed between researchers from Croatia, Serbia and Slovenia. The basic nodes were: “sleeping habits”, “eating habits”, “physical activity”, “time in front of the screen” and other (new) habits.

More detailed analysis of empirical material within nodes:

- sleeping habits (stays the same, goes to bed later, sleeps more);
- eating habits (eating habits are related to emotions (eat more, unhealthy), eat less, eat healthier, eat carefully when it comes to selection of foods, no changes in eating habits);
- physical activity (PA) leads to more sub-responses (less PA, more PA, new PA program);
- time in front of the screen (more time in front of the screen (TV, mobile phone, tablet), for work, study, information or entertainment);
- New habits/routines (new, re-established or transferred habits).

### Sleeping habits

Sleeping, as one of the basic physiological functions of human beings, plays an important role in the process of learning, memory, work results and the overall quality of life of the student population (27). Research results show that sleep-related variables, such as lack of sleep, sleep habits/schedules, and the like, affect student performance and interpersonal relationships (28–30). As epidemiological measures caused by the COVID-19 pandemic significantly increased the time respondents spent at home, it was necessary to investigate their habits/sleep schedule.

Mann Whitney *U* test found that there was no statistically significant difference between the differences (deltaQ2a) in “bedtime” between the analyzed study program groups for female students (W = 046060.5, *p*-value = 0.358) and male (W = 18,389.5, *p*-value = 0.986).

The variable (deltaQ2b) - “wake-up time” showed a significant difference between study program groups in the student subsample and it was found that there is a statistically significant difference (W = 20,742.5, *p*-value = 0.028) but not in the female subsample (W = 47,785.5, *p*-value = 0.089).

The variable (deltaQ3) - “sleep time” did not show statistically significant differences between the study program groups of female students (W = 44,736.5, *p*-value = 0.752 nor in male students (W = 17,076.0, *p*-value = 0.225).

Qualitative analysis showed that more than half of the students observed changes in sleep pattern and sleep quality that were not detected as statistically significant by quantitative research, and in some cases we detected sleep disorders, as evidenced by students' responses to sleep pattern.

“I definitely prolonged my going to bed time for 3 h, so instead of going to bed at midnight, I went to bed between 2 and 3 am in the morning, because I could get up later.” (Student, social sciences).

“I have problems with my sleep, I go to therapy every year and I have periods when everything is fine and I have periods when sleep is a disaster. I even took medication for it, but after those 1st months of COVID-19 it didn't matter anymore or I didn't even pay attention to what time it was, or what time of day it was, I stayed awake until 5 in the morning, actually complete chaos.” (Student, social sciences)

“No, there is basically no change because I get up at 6 am and go to bed from 10 pm to 11 pm for a year, or more than a year, and during the pandemic it was more or less the same. Nothing has changed, I slept the same.” (Student, sports)

“Sleeping at 10 pm, I leave the phone much earlier, I really liked it, I could have fallen asleep earlier because I didn't look at the screen, I didn't answer some messages, I would finish something quickly, leave the phone like that and rest.” (Student, sport)

Students' reactions point to a certain transformation of everyday life practices in two ways: students who talked about longer sleep said it was due to the fact that “they had nothing else to do” or the fact that their daily life at faculty is simply full of obligations. Longer sleep was *necessary* to finally get some rest. The students (most from SPG1), who reported going to bed at the same time, said they “tried to maintain a routine”, meaning that in the face of epidemic measures caused by the COVID-19 pandemic, they had to make an effort to maintain more or less the same routine of their daily life.

### Sedentary behavior and physical activity of students

Physical inactivity is considered to be the fourth leading risk factor for mortality in the world and is estimated to result in 3.2 million deaths each year (31). The risk of increased physical inactivity was higher with the introduction of emergency epidemiological measures to prevent the spread of COVID-19. These measures included the closure of gyms, fitness centers, the closure of parks and outdoor gyms, as well as a ban on gathering more than five people at the same time, in all three countries. At the same time, these measures directly affected freedom of movement, but also the ability to engage in physical activity (12, 14, 32–34). In this regard, this research points to the characteristics of students' physical activity during the quarantine of the first wave of the coronavirus pandemic.

Analysis of differences in the inactivity difference variable (deltaQ4) indicates a statistically significant difference in the inactivity time of female students of the analyzed study program groups (W = 50,505, *p*-value = 0.003), also between male students in SPG1 and SPG2 (W = 21,298.5, *p-*value = 0.007). The time of inactivity is significantly lower in the subsample of students in the study program group 2, as the restriction of sports activities affected members of this group less than the students of SPG1, which increased the time of inactivity (on average by almost an hour), which is a logical consequence of higher PA before SPG2.

Analysis of the variable differences in walking for transport (deltaQ10) indicates a statistically significant difference in time spent walking for transport and recreation between study program groups of female students (W = 38,512.5, *p*-value = 0.012) but not between male students (W = 17,998, *p*-value = 0.733). Walking for transport and recreation decreased in both subsamples. Female students of SPG1 group reduced (SPG1 median−60) walking time for transport and recreation more than female students of SPG2 (SPG2 median−40), while students of both groups obviously increased (SPG1 / SPG2 median = −40) walking for transport and PA, which may be the result of more time devoted to recreational walking.

The difference in time spent in sports activities (deltaQ11) between the analyzed study program groups is also statistically significant. The difference was also found in the subsample of female students (W= 32,678, *p*-value <0.001) and male students (W = 14,945, *p*-value = 0.002).

It can be seen that, in general, SPG1 students significantly reduced the time spent in sports activities (median−30) compared to students of SPG2 study program groups (median 0). The same difference was found in the subsample of SPG1 female students (median−30) compared to SPG2 female students (median 0).

The variable (deltaQ13) – “time of moderate or vigorous physical work” does not indicate statistically significant differences between the study program groups SPG1 and SPG2 female students (W = 46,255.5, *p*-value = 0.290) and male (W = 19,753, *p*-value = 0.184).

Interviews also confirmed some results of quantitative analysis in changing time for physical activity (PA). Here are some student responses that explain the reasons for lower exercise intensity, mainly due to movement limitations, related to laziness, lack of discipline, and time required to adjust:

“A lot has changed. I am quite active, I like to walk, but when quarantine was introduced, we were all forced to be in the house and then you could not move, so we were all pretty lazy and it is certain that we all gained a little weight, including me... Simply, sometimes you don't feel like cooking, so order food delivery, and since you're so stressed out, it had a bad effect on my physical activity.” (Student, humanities)

“It was a disaster, literally, although I am the type of person who prefers to be at home and I am not particularly physically active during the day, I mostly sit and study, rest or possibly go to the gym which is my only type of physical activity (...) I eat everything and anything, not taking care of myself, not training, not disciplined and it all started to annoy me.” (Student, social sciences)

“Maybe the first month there was less exercise because I didn't have the riding and fitness training I normally had. Then, after a month, I replaced exercising in the gym with exercising at home, which is not the same thing.” (Student, sports)

However, half of the students (15) trained regularly, and some even increased their training intensity. In the second half, there were students who were less physically active (6) and equally physically active (8). Some of them stated that they exercise at home, through online training sessions or individually. One student (sports science) stated that after 31 days of quarantine, he became first a coach to his mother and later an online coach to friends and relatives. Several students reported lower PA levels because they were active athletes (e.g., football, horseback riding), due to an emergency measures they were forced to stop training in at least the first 2 weeks of quarantine. On the other hand, several students stated that they are more physically active than usual. Some sports students said exercise, regular sleep and healthy eating habits are linked. One student (social sciences) started training only after the introduction of emergency measures because she gained a few kilograms due to an unhealthy diet during the pandemic. Students of the Faculty of Kinesiology (KIF), Faculty of Sports and Physical Education (FSFV) and Applied Kinesiology (AK) state that they exercised regularly, which again leads to the conclusion that students of these study programs have more clearly defined health-related routines in terms of daily practices.

“I exercised at home, before I went to the gym 3 times a week (...) After the first week of quarantine I was completely crazy because I was used to going out, hiking, playing volleyball during the summer, I missed it so much, I felt captured. Then I bought the online program HIT (high intensity training)... from a kinesiologist, just to get an idea, because I know how to exercise on my own, but that's just to have an idea, as well as a little incentive, and it really came in handy (…) I'm practicing now every day.” (Student, sports)

“I tried to stay physically active and every day I did whatever came to my mind, from running, Pilates, kickboxing, we even ordered a punching bag and hung it on a tree in the yard, so I always had some kind of recreation, activities; I read what I like.” (Student, sports)

“I practiced regularly. Then my mom started exercising with me in the living room. When my aunt heard that my mother was training, then she also wanted to train with us, so we had to use Skype. Then my girlfriend wanted to practice with us, and so did her father, so my brother asked for the same. At one point, 5–6 of them practiced. So, I practiced and became an online coach (laughs).” (Student, sports)

Having in mind the overall results of quantitative and qualitative data, we can conclude that students were generally less physically active during the 2-month epidemiological measures. Not only did exercise as a physical activity decrease, but daily walking also decreased (not significantly in relation to gender and study program) as a result of the “*lockdown*.” All students reported a decline in walking (40 to 50 min per day), with the largest decline in sports students (SPG1) and fewer in students of SPG2 group.

Such results were also expected, as the quarantined state suddenly closed entire families to apartments with limited time and space to move and exercise. On the other hand, the narrative responses of students showed that the effect of restrictive measures results in increased inactivity, less walking, while exercise increases in several students who were previously inactive and use *lockdown* to start exercise. Additionally, students with an active lifestyle and regular physical activity habits found a way to continue exercising at home, despite space constraints. This also points to the fact that emergency epidemiological measures have necessarily influenced the change in students' daily practices, but also that they have produced some new strategies that some students have implemented to stay or even become more physically active.

### Screen time as students' daily habit

Time spent on screens as a passive, sedentary habit, changed the most during the study period due to the shift of reality to online mode.

Analysis of the variable time difference spent in front of the screen (deltaQ6) does not indicate a statistically significant difference between study program groups SPG1 and SPG2 subsample of female student subsample (W = 41,896, *p*-value = 0.329) and male student subsample (W = 18,255, *p*-value = 0.977).

This is expected, as the increase in time spent in front of the screen can be seen as a result of the increased need to use technical aids (computers, tablets, smartphones) to conduct online forms of study and continue education, which was significant in both groups of students.

One of the segments of the analysis, which is manifested through the analysis of the variable differences in time spent in front of computers (deltaQ7c), partially determines its impact on differences. A statistically significant difference was observed between the two study program groups of female student subsamples (W = 36,448, *p*-value < 0.001) and male student subsamples (W = 15,392, *p*-value = 0.006). There is a visible increase in time spent in front of computers in subsamples of male and female students of study program group 2 (SPG2 students median = 2, SPG2 students median = 2) compared to medians of subsamples of male and female students of study program group 1 (SPG1 students median = 1.5, SPG2 students median = 1).

Statistically significant differences were also observed in the analysis of the variable differences in time spent in front of the screen to meet study obligations (deltaQ8a) between study program groups of female student subsample (W= 33,297.5, *p*-value < 0.001) and male student subsample (W = 15,963.5, *p*-value = 0.026).

The SPG2 male student subsample increased screen time for study more than SPG1, (SPG1 median = 0.5, SPG2 median = 1), as well as female students, where time spent in front of the SPG2 screen changed significantly compared to SPG1 (SPG1 median = 1, SPG2 median = 2).

The results of the interviews confirm the obvious increase in time spent in front of screens and sedentary time, mainly as a result of increased time for learning and other study obligations.

“Yes, it only increased... records were broken, there was no other, because I was writing a master's thesis. It was 5, 6 h a day, and then the phones, but yes, there was certainly more, much more in front of the screen, a lot of sitting, it was even boring, quite boring.” (Student, sports)

“The time spent on the screens, however, has increased abnormally. I was practically at the computer all day long. First because we had lectures, and then we played games and that was the only way you could hang out with someone and talk online, otherwise it would be absolutely pathetic (...) Yes, and time spent on a cell phone, I was on the phone all the time... both on the computer and on the phone together.”(Student, social sciences)

“Watching movies and series has definitely increased, I know the first 10 days, it was 24 h a day (…) When I did not have any ideas for movies, then I asked someone to recommend a new movie to me. (…) Over time, it was less and less. The second month was more focused, I think there were still movies and series, but a lot less. Then the lectures started somehow, and I kept pace with them.” (Student, social sciences)

In addition, the interviews confirmed that “time spent in front of screens” appears to be the dominant daily habit. All students have in common that they spend a lot of time in front of screens. However, their answers should be classified into three groups:

- passive time spent watching television/mobile phone;
- time spent in front of the screen due to online learning/studying;
- time spent on social media to access information on the COVID-19 pandemic and/or entertainment.

These responses suggest an association between physically inactive behavior and time spent in front of (different) screens, which was also confirmed in basic research (12). Increased physical inactivity is closely related to increased time spent behind screens, such as a TV, smartphone, computer, or tablet. In the primary sample, 65% of the increased time spent on screens, along with increased meals, unhealthy food consumption, and decreased exercise, was explained by an increase in body weight in 20.6% (12).

### Eating habits and other habits

Reported quantitative and qualitative data indicate more inactive, sedentary behavior, more time spent on screens and situations related to different emotions (fear, loneliness). These emotions were associated with eating more of fast and unhealthy food in one third of students (female, humanities / social sciences or natural sciences), mostly at the beginning (first 2 weeks) of the introduction of emergency epidemiological measures, which resulted in weight gain. A disrupted work/study routine resulted in unstructured schedules, which could lead to boredom and/or increase time spent on screens, which in turn can lead to overeating and consequent unbalanced energy intake (35).

The analysis did not identify statistically significant differences in the variables of differences between groups SPG1 and SPG2 and subsamples of male and female students, related to eating habits and other habits (for variables regular meals (Q17a), unhealthy food consumption (Q17c), alcohol (Q17d) and smoking (Q17e).

Regular meals (Q17a) generally for SPG1 and SPG2 no statistically significant differences were observed between female student subsamples [H_1_ = 3.281, *P* = 0.070] and male student subsamples [H_1_ = 0.208, *P* = 0.649]. As well as regular meals and consumption of unhealthy food (Q17c) was not differ among students of different study program groups of male student subsamples [H_1_ = 0.086, *P* = 0.769] or subsamples of female student [H_1_ = 0.243, *P* = 0.622]. It can be interpreted that belonging to different study programs does not show an impact on eating habits.

The variable alcohol consumption (Q17d) did not show statistically significant differences between the study program groups of female student subsamples [H_1_ = 0.018, *P* = 0.894], but a marginally statistically significant difference was observed in the male student subsample [H_1_ = 3.741, *P* = 0.053], which speaks of reducing the amount of alcohol consumption by students, which can be explained by the decline in socializing of young people, often accompanied by alcohol consumption.

**Figure 1:**
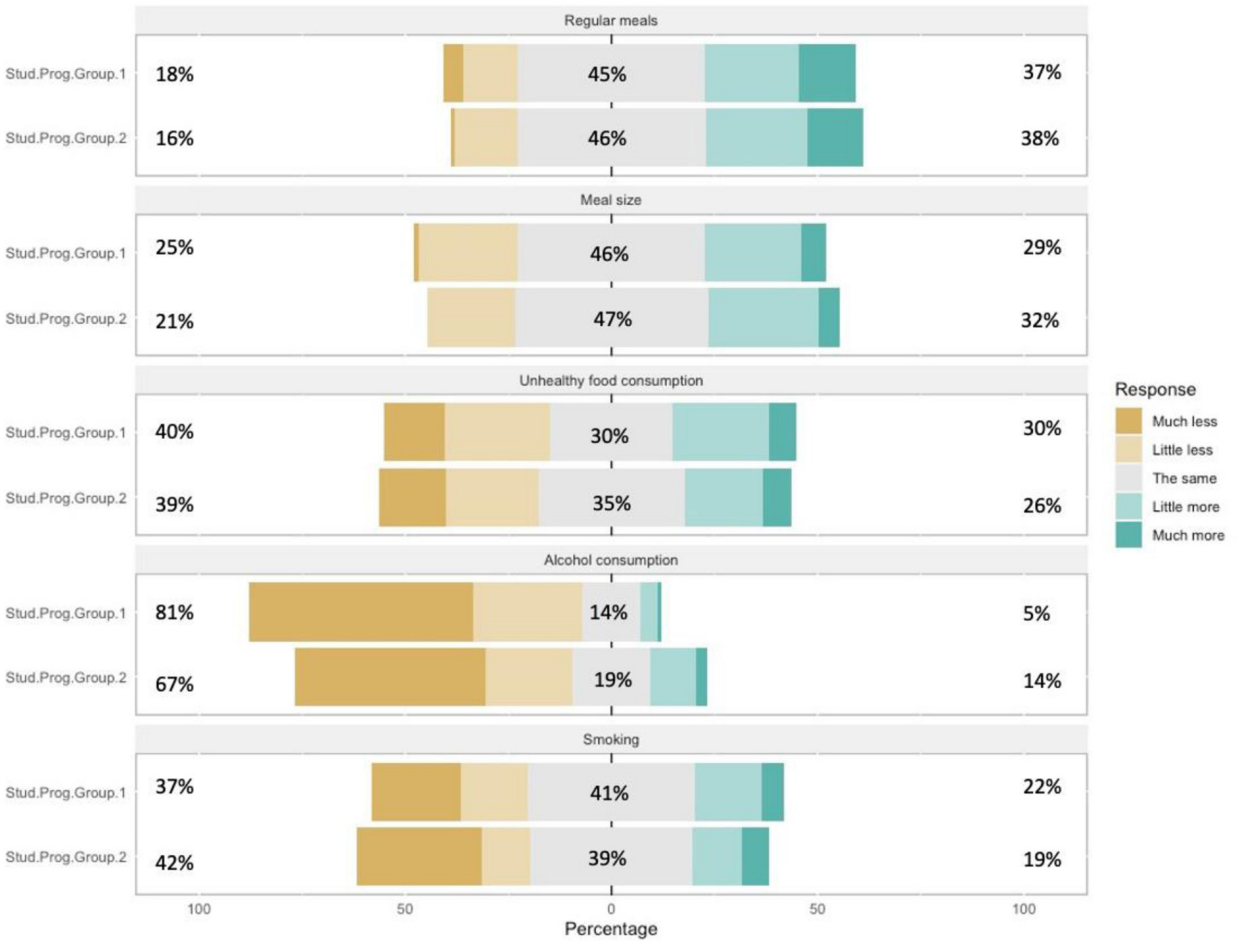
Changes in eating habits and other habits – female students.

**Figure 2:**
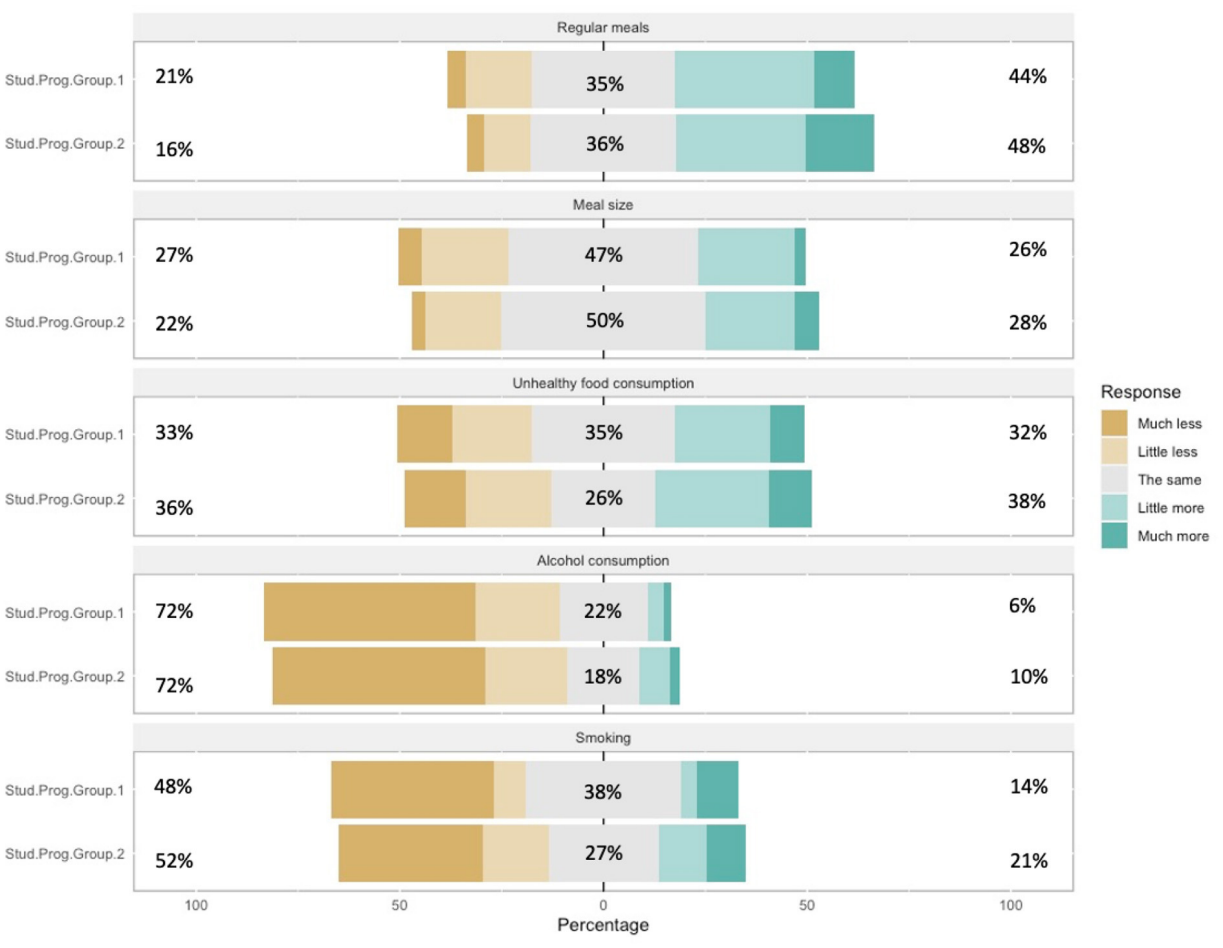
Changes in eating habits and other habits – male students.

Likewise, smoking (Q17e) as a health risk behavior showed a statistically insignificant difference between the study program groups of female student subsamples [H_1_ = 0.151, *P* = 0.697] and study program groups of male student subsamples [H_1_ = 0.427, *P* = 0.513] (see Figures 1, 2).

Qualitative analysis shows some quantitative results regarding the question of how feeding habits have changed negatively:

“For the first 2 weeks, we just ordered food. By ordering again and again, there is e.g., lack of vegetables… Lack of some healthier foods… There are no specifically made meals such as breakfast, lunch, dinner, but it is more like, lunch is at 12 and then it is a meal for the whole day because we eat so much… And then dinner at 10 in the evening, which is completely disturbed.” (Student, social sciences)

“It wasn't a big habit, but I liked to eat a much richer dinner in the evening, a little sweet during the day, so that it also accumulates there – I gained two kilos, let's say…” (Student, sports)

“I ate… I think I definitely ate more, that made me feel bad, more sweet, so much unhealthy food, but because it was some new environment, I didn't know so many products on the market, and because my boyfriend and I had a different diet, what suits his stomach, may not suit mine. So it took me a while to figure out what I was doing for myself at home, to adjust to that environment and start, let's say, eating better. Less in the evening, because I feel like I've eaten a full meal already; I was very tired and suddenly I had a greater need for sweets. So, over the last few weeks, I've been slowly fixing that. Because I was somehow stricter, I ate more carefully.” (Student, science)

It is encouraging that 2/3 of the students in the interviews stated that they did not change their eating habits or changed them in a positive way, with a healthier, more balanced diet and regular meals.

“I cooked every day to help out and then I found some recipes, meals that can be prepared a little healthier, a little less fatty, and then I found a way and food that suits me and (…) Then I started to establish a rhythm that suits me. Then, at one point, I felt really good, I got up in the morning and that day I knew I was going to eat what I like, I was going to eat something sweet, I was not going to bother about it and I was just pretty relaxed, I got out of that cramp, actually during the extraordinary measures, it helped me a lot to calm down, relax, to understand some things differently, it helped me.” (Student, sports)

This new daily rhythm caused students to acquire some new habits/skills or to “strengthen” the intensity of some old behaviors. Several female students said they have started cooking healthier food, while others more sweetscakes. Although cooking is a common answer, we should classify these answers into two groups: preparing healthy meals and cooking and baking desserts. Some students began to learn foreign languages or develop their talents for drawing or photography. These answers point to the fact that the interviewed students found some strategies for *surviving* the new everyday life. One of the indicators is the redirection of *excess* free time to the acquisition of new knowledge and skills.

“I started learning Japanese more intensively. (…) Basically, this quarantine was an opportunity for me to focus a little more.” (Student, sports)

“I started doing meditation and breathing exercises. Now I am more relaxed and believe in myself. That was the biggest change. Yes, this meditation relaxed me and gave me a better sleep.” (Student, sports)

“(...) On the one hand, it was great to learn a little more, reading things that I am interested in…” (Student, mechanical engineering)

In addition, unhealthy habits such as alcohol consumption and smoking before and during the pandemic did not show statistically significant differences between the sexes and study programs, but a decline was observed in students representing 70.42% of the population (average = 1.92 on Likert scale) in alcohol consumption and smoking (34.98% of the student population) and slightly lower smoking was also observed during emergency measures. The results coincide with a general reduction in alcohol consumption and smoking (12) and can be explained by severe limitations in social life and accompanying social habits, drinking and smoking (especially among young people), including countries where a pandemic has broken out.

“Since there were no parties at that time, I can easily say, there was not so much alcohol, all in all, it was gloomier, more serious.” (Student, sports)

In addition, most students described the time they spent with family members as a “new routine” and generally described it in a positive context.

“I think that by shutting people down, they had to look at their family members to see if everything was okay with them. And then people probably turned to themselves and their own, which is most important because they realized how important these relationships are. I've improved relationships with my family, I think, and I've had a really good relationship with them before, especially with my mom and sisters. We realized that we love our Sunday lunch when we are not limited by time, that we enjoy how much we love spending time together.” (Student, sports)

“I think the only positive thing is that some people who work a lot have finally spent more time with their family and made up for it with them, so that's one of the positive things.” (Student, humanities)

The results of the qualitative research show that the unforeseen social crisis, conditioned by the threat to human health, has led the respondents to reconsider their daily life priorities, rhythm of life and family life. For many of them, this is the first extreme life situation, and they were somewhat *forced* to look for new strategies to maintain the old everyday life or establish a new routine. One of the new routines was the obligation to return home (due to the closure of dormitories and the closure of state borders for foreign students) and stay all day with family members. Given most of the interlocutors' responses, this was also one of the few positive changes during quarantine.

## Discussion

Taking into account the results of previously conducted quantitative research, we can highlight several negative consequences caused by the introduction of emergency measures caused by the COVID-19 pandemic, namely: poor mental health with symptoms of post-traumatic stress disorder, avoidance of other people and anger, fears, frustrations, boredom, stigmatization, lack of stocks, lack of adequate information, financial loss (36–40), as well as an increase in negative health impacts and routines such as reduced physical activity (walking, exercise), in favor of increased sedentary behavior, mainly due to the time people spend in front of screens (12, 41). More precisely, from the perspective of the results of our study, we can summarize the impact of emergency epidemiological, i.e., restrictive measures caused by the COVID-19 pandemic in two directions. First of all, the negative consequences of the “lockdown” are related to the development of unhealthy daily habits in the student population, as follows:

- reduction of physical activity,
- increasing inactive time spent in front of the screen,
- unhealthy diet associated with overeating or “emotional overeating”, especially food that provides *comfort* due to boredom,
- Interrupted scheduling and due to possible psychological factors (uncertainty, loneliness and fear) that resulted, according to the testimonials in qualitative research, weight gain of some respondents or sometimes sleep disorders.

These negative consequences should not be ignored when it comes to the health of the population, especially young people. Particular attention should be paid when similar emergencies occur with a more sensitive view of government policy when preparing measures such as closing education systems, sports and recreational programs to avoid serious impacts not only on physical but also on mental health.

The results of the research also indicate some positive outcomes of the 2-month pandemic experience, namely:

- Some students improved their lifestyle, ate healthier during the pandemic, and had more time for hobbies and the acquisition of new knowledge and skills (learning, reading, meditation, acquiring new skills);
- It is significant that students of sports and medical study programs spoke about maintaining daily routines and health habits even during a pandemic. In this regard, they rarely talked about sleep disorders, and more often about healthier eating, maintaining a routine of continuous physical activity adapted to new circumstances. These habits have a protective role against stress in challenging times because the individual can better guard against new, unpredictable, or threatening stimuli (42);
- SPG 1 students confirmed the importance of developing habits and routines that enable effective response to emergencies that threaten the routine of daily life.

Results we obtained are to some extent in line with Bauman's “liquid life”, which we have used as theoretical framework of the research. As we assumed, the “old” balance between freedom and security was lost for the first time in our respondents' and interviewees' lives. The *new* epidemiological and social circumstances turned their life into a state of concern about the *invisible* danger. Nevertheless, the results indicate that SPG1 adopted a more successful strategy to fight against the mentioned *invisible danger*.Such selected answers point to the double consequences that extraordinary measures have left on the everyday life of university students, with special emphasis on sleeping habits, diet, sedentary and physical activities, and time spent in front of screens. Finally, both negative and positive consequences of the above-mentioned measures indicate individual *strategies* used by university students for confronting with unforeseen epidemiological as well as socio-psychological circumstances.

## Conclusion

Taking into account the results of this field research, we can conclude that changes in daily life routines in the student population were obvious. During the *lockdown*, students spent most of their time at home and were mostly inactive. They lacked socializing, interpersonal communication, social interaction and free movement, which they mostly made up for with more time spent on social media – in front of the screen and sedentary. By summarizing the impact of the COVID-19 pandemic measures on students' daily lives, by showing changes with positive and negative impacts, we were able to explore students' social responses and their adaptation to new social circumstances. Although the negative impact is primarily related to negative health habits in the domains of physical (in)activity, time spent in front of screens and consuming unhealthy food, they positively affected partnerships and time needed for self-development of students. Students of sports study programs showed a more positive response and successful coping with the new situation compared to students of other study programs, which confirms the importance of maintaining daily routines and habits in times of extreme situations such as the COVID-19 pandemic. Finally, by researching the daily life practices of students of the three societies during emergency, extraordinary epidemiological measures, this research contributes to illuminating and understanding the social consequences of these measures on young, healthy and highly educated social actors. It also provides a deeper insight into the day-to-day *strategies* they are undertaking to counter or adapt to the “new normal”. However, it should be borne in mind that the research was conducted in the middle and at the end of the first wave of the COVID-19 pandemic. At the time of writing, these societies were experiencing a third wave of pandemics. Therefore, it is important to take into account the need to continue research (transformation) of everyday practices of the student population.

## Data availability statement

The raw data supporting the conclusions of this article will be made available by the authors, without undue reservation.

## Ethics statement

The studies involving human participants were reviewed and approved by Ethical Committee of the Faculty of Sport and Physical Education, University of Novi Sad, Serbia (46-11-07/2020-1). All respondents and interviewees signed written informed consent for participation.

## Author contributions

SP, IM, DK, SB, and SR wrote the manuscript, performed analyses, and revised the manuscript. SP, IM, and SB collected the data. SP, IM, DK, and SR overviewed previous studies. SP, IM, and DK discussed the results. All authors contributed to the article and approved the submitted version.

## Funding

This study was financed by the authors' institutions: Science and Research Centre Koper, Slovenia, research programme Kinesiology for the Quality of Life (P50381), funded from the Slovenian Research Agency, as well as Faculty of Sport and Physical education, University of Novi Sad, Serbia under the project Developing Future Sports Entrepreneurs in Europe: University Education to Promote Entrepreneurial Intentions in Sports Science Students (142-451-2596/2021), financed by the Provincial Secretariat for Higher Education and Scientific research.

## Conflict of interest

The authors declare that the research was conducted in the absence of any commercial or financial relationships that could be construed as a potential conflict of interest.

## Publisher's note

All claims expressed in this article are solely those of the authors and do not necessarily represent those of their affiliated organizations, or those of the publisher, the editors and the reviewers. Any product that may be evaluated in this article, or claim that may be made by its manufacturer, is not guaranteed or endorsed by the publisher.
